# Case report of a recurrent resected glucagonoma

**DOI:** 10.1016/j.amsu.2022.103604

**Published:** 2022-04-09

**Authors:** Ouadi Yacine, Feryel Letaief Ksontini, Ahmed Ben Mahmoud, Houcine Magherbi, Samir Fadhel Fterich, Montasser Kacem

**Affiliations:** a Department of Surgery, A La Rabta, Tunis, Tunisia; b Faculty of Medicine of Tunis, Tunis El Manar University, Tunis, Tunisia

**Keywords:** Case report, Glucagonoma, Neuroendocrine tumor, General surgery

## Abstract

**Introduction and importance:**

Glucagonoma is a rare neuroendocrine tumor (NET). Most glucagonomas are in the tail or body of the pancreas and are diagnosed at a metastatic stage. We report a case of an early recurrence after surgical resection of a glucagonoma and its management.

**Case report:**

We present a case of a 44-year-old female patient with no medical and surgical history, operated on in May 2018 for pancreatic glucagonoma revealed by skin necrolytic migratory erythema. The patient was regularly monitored by clinical exams and CT scans. In December 2020 (31 months postoperatively), we noticed the recurrence of the cutaneous lesions.

Admission laboratory measurements demonstrated hyperglycemia as well as elevated blood Glucagon levels. Explorations showed 3 retro-pancreatic lesions. Based on these findings, we concluded that it was a recurrence of her glucagonoma. The patient was operated on by median laparotomy We performed a warshow's procedure.

Pathology confirmed the endocrine nature of the 3 nodules. We are currently 6 months behind the surgery. The examination is strictly normal with no recurrence of the skin lesions so far.

**Clinical discussion:**

Surgical resection on a recurrent glucagonoma is what is unique in our case as we haven't found any case in the literature to our knowledge.

What is also unique about our case is both the local aspect of the recurrence and the multiplicity of the tumors observed as multiple nodules around the tail of the pancreas. These lesions were not metastatic lymph nodes as confirmed by pathology. Probably it was an effraction of the big tumor at enucleation.

**Conclusion:**

Due to its rareness, there is no clear consensus on the management of glucagonomas therefore we chose to write our case in order to further enrich the literature to achieve one-day guidelines for glucagonomas treatment.

## Highlights

• Glucagonoma is rare.
• NME is considered as an important symptom.
• Surgery is the only permanent solution.
• Recurrence is frequent therefore follow up is important.

## 1 Introduction

Glucagonoma is a rare neuroendocrine tumor (NET). Most glucagonomas are in the tail or body of the pancreas and are diagnosed at a metastatic stage. We report a case of an early recurrence after surgical resection of a glucagonoma and its management.

This case report has been reported in line with the SCARE Criteria [1].

## 2 Case presentation

We present a case of a 44-year-old female patient with no personal medical and surgical history nor family history of multiple endocrine neoplasia, operated on in May 2018 for pancreatic glucagonoma revealed by skin necrolytic migratory erythema. The patient had a simple enucleation of the mass. Pathology concluded to an intermediate grade (G2) pancreatic neuroendocrine tumor with a Ki67 at 7%. The postoperative period was uneventful, and all cutaneous lesions disappeared.

The patient was regularly monitored by clinical exams and CT scans. In December 2020 (31 months postoperatively), we noticed the recurrence of the cutaneous lesions.

On admission, she had a good general condition, stable vital signs, no abdominal mass but with skin findings suggestive of necrolytic migratory erythema.

Admission laboratory measurements demonstrated hyperglycemia (1.3 g/L, normal values 0.7–1.1 g/L) as well as elevated blood Glucagon levels (>150 pmol/L, normal value < 60 pmol/L). The serum levels of Chromogranin A, were normal (31ng/ml).

MRI and CT-scan showed 3 retro-pancreatic lesions of 10, 13, and 16 mm in diameter. The octreotide scan was normal. Based on these findings, we concluded that it was a recurrence of her glucagonoma.

**Fig. 1:**
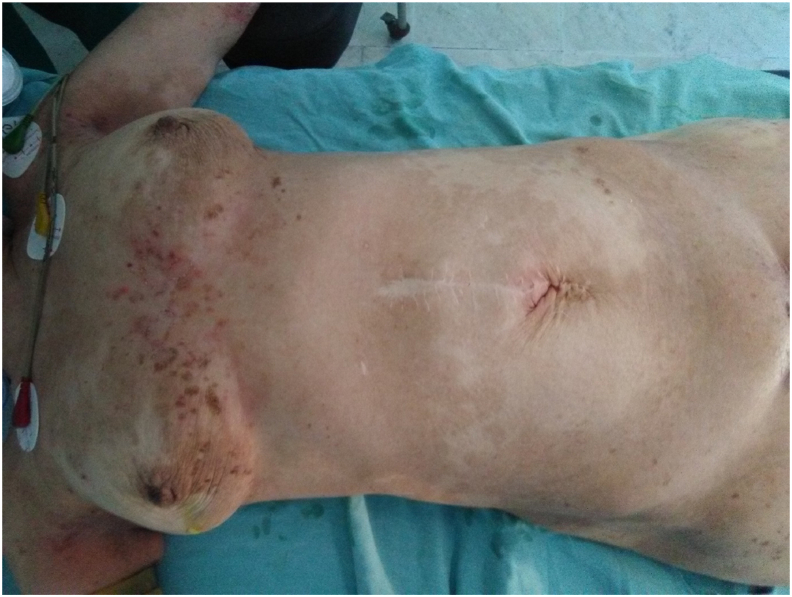
In operative photo showing the NME skin lesions.

**Fig. 2:**
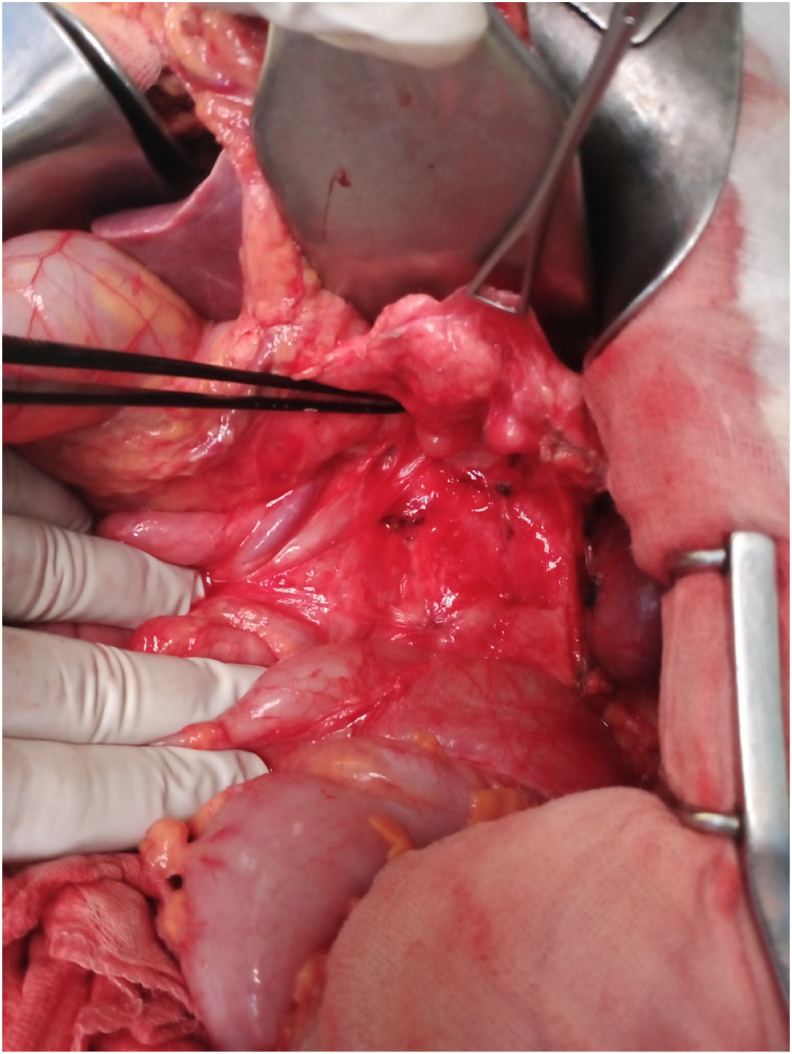
In operative photo of the 3 pancreatic lesions found after posterior dissection of the pancreas.

The patient was operated on 28-05-2021 by an attending surgeon at our department by median laparotomy (Fig. 1). On exploration, no ascites, no hepatic metastases. After dissection of the left part of the pancreas, the 3 nodules described upper are found below and behind the tail of the pancreas varying from 10 to 15 mm in diameter (Fig. 2).

**Fig. 3:**
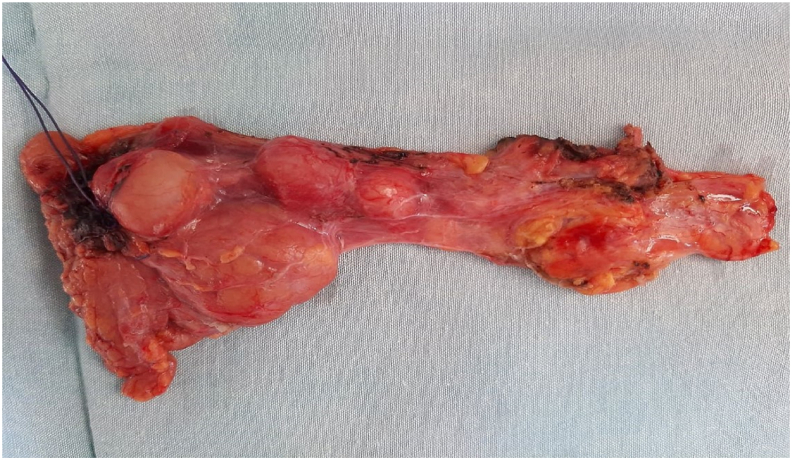
Post operative photo of the resected left pancreas showing the posterior lesions.

We performed distal pancreatectomy with spleen preservation and resection of the splenic vessels as shown in Warshow's technique (Fig. 3). The postoperative period was uneventful, and all cutaneous lesions disappeared. The patient was very pleased with the result and was willing to comply with the follow-up regimen.

Pathology confirmed the endocrine nature of the 3 nodules and concluded to an intermediate grade (G2) pancreatic neuroendocrine tumor with a Ki67 at 5%. We are currently 6 months behind the surgery. The patient has no functional complaints. The examination is strictly normal with no recurrence of the skin lesions so far.

## 3 Discussion

Glucagonoma is a rare neuroendocrine tumor (NET), with an incidence of one in 20 million people. More recent reports suggest that they represent 2–7% of pancreatic neuroendocrine tumors. Most glucagonomas are in the tail or body of the pancreas [2].

As it is pointed out in our case the necrolytic migratory erythema is a common symptom but lacks specificity which led to a late discovery of this tumor. Indeed, our patient was explored for chronic dermatitis for 6 years before the final diagnosis was found. This is concurring with the literature as a recent series found a median time of 39 months from onset of symptoms to diagnosis [3].

What we find interesting in our case is the early recurrence of the resected glucagonoma.Indeed, our patient underwent surgery one year prior to its transfer to our department. The patient had a history of enucleation of a 14cm glucagonoma. The surgery, in this case, was insufficient as it is well known that enucleation is only indicated in small NET's [4]. Therefore, recurrence was an inevitable evolution. That is precisely why we opted for a more radical approach by performing a Warshow's procedure.

Surgical resection on a recurrent glucagonoma is what is unique in our case as we haven't found any case in the literature to our knowledge. The only reported case of recurrent glucagonoma wasn't resected due to vascular considerations. Other reported cases of recurrence were of metastatic glucagonoma [5].

What is also unique about our case is both the local aspect of the recurrence and the multiplicity of the tumors observed as multiple nodules around the tail of the pancreas. These lesions were not metastatic lymph nodes as it was confirmed by pathology. Probably it was an effraction of the big tumor at enucleation.

To confirm our curative procedure, clinical assessment and dosage of endocrine markers should be practiced. We will also perform CT scans every 3–9 months as suggested in the 2016 ENET's consensus guidelines.

## 4 Conclusion

Due to it’s rareness there is no clear consensus in the management of glucagonomas therefore we chose to write our case in order to further enrich the literature to achieve one day guidelines for glucagonomas treatment.

## Ethical approval

There is no ethical committee in our country (Not applicable for this manuscript).

## Sources of funding

No sources of funding.

## Author contribution

All authors contributed to the study design.

## Declaration of competing interest

All authors declare that they have no any conflicts of interest.

## Registration of research studies

1. Name of the registry:
2. Unique Identifying number or registration ID:
3. Hyperlink to your specific registration (must be publicly accessible and will be checked):

## Guarantor

Ouadi Yacine.

## Consent for publication

Written informed consent was obtained from the patient for publication of this case report and any accompanying images. A copy of the written consent is available for review by the Editor-in-Chief of this journal on request.

## Provenance and peer review

Not commissioned, externally peer reviewed.
